# CULTURALLY TAILORED INTERVENTION EFFICACY TO PROMOTE BREAST CANCER SCREENING AMONG AMERICAN INDIAN WOMEN

**DOI:** 10.1093/geroni/igad104.2714

**Published:** 2023-12-21

**Authors:** Soonhee Roh, Yeon-Shim Lee, DenYelle Kenyon, Amy Elliott, Daniel Petereit, Anu Gaba

**Affiliations:** University of South Dakota, Sioux Falls, South Dakota, United States; San Francisco State University, San Francisco, California, United States; University of South Dakota, Sioux Falls, South Dakota, United States; Avera Research Institute - Sioux Falls, Sioux Falls, South Dakota, United States; Rapid City Regional Hospital, Rapid City, South Dakota, United States; Sanford Roger Maris Cancer Center, Fargo, North Dakota, United States

## Abstract

**Purpose:**

The purpose of this project was to develop and evaluate the feasibility and effectiveness of the Mobile Web App Breast Cancer Screening (wMammogram) intervention among American Indian (AI) women. Method: Using a randomized controlled trial design, 122 AI women (40-70 years old) were recruited and randomly assigned to the wMammogram group (n=62) to receive culturally and personally tailored multimedia messages through a mobile web app along with health navigator services or the control group (n=60) to receive a printed educational brochure. Outcome measures included mammogram receipt, intention to receive breast cancer screening after the intervention, and satisfaction with and perceived effectiveness of the intervention. Result: A significantly higher proportion of women who received the intervention (42%, 26/62; p<.01) completed mammograms by the 6-month follow-up compared with the control group (20%, 12/60). The wMammogram group, compared with the control group, reported significantly higher ratings on perceived effectiveness of the intervention (t120=-5.22, p<.001), increase in knowledge (t120=-4.75, p<.001), and satisfaction with the intervention (t120=-3.61, p<.001). The wMammogram group also expressed greater intention to receive a mammogram in the future when it is due (100%, 62/62 vs. 85%, 51/60) and were more willing to recommend the intervention they received to their friends (98.4%, 61/62 vs. 90%, 54/60) compared with the brochure group. These differences were statistically significant.

**Conclusion:**

Based on our results, a mobile web app-based intervention combined with health navigator service was a feasible, acceptable, and effective intervention mechanism aimed at promoting breast cancer screening in AI women.

